# Age- and sex-specific differences in immune responses to BNT162b2 COVID-19 and live-attenuated influenza vaccines in UK adolescents

**DOI:** 10.3389/fimmu.2023.1248630

**Published:** 2023-10-06

**Authors:** Cecilia Jay, Emily Adland, Anna Csala, Nicholas Lim, Stephanie Longet, Ane Ogbe, Jeremy Ratcliff, Oliver Sampson, Craig P. Thompson, Lance Turtle, Eleanor Barnes, Susanna Dunachie, Paul Klenerman, Miles Carroll, Philip Goulder

**Affiliations:** ^1^ Nuffield Department of Medicine, University of Oxford, Oxford, United Kingdom; ^2^ Department of Paediatrics, University of Oxford, Oxford, United Kingdom; ^3^ Wellcome Centre for Human Genetics, University of Oxford, Oxford, United Kingdom; ^4^ Peter Medawar Building for Pathogen Research, University of Oxford, Oxford, United Kingdom; ^5^ Division of Biomedical Sciences, Warwick Medical School, University of Warwick, Warwick, United Kingdom; ^6^ Institute of Infection, Veterinary and Ecological Sciences, University of Liverpool, Liverpool, United Kingdom; ^7^ National Institute for Health and Care Research (NIHR) Oxford Biomedical Research Centre, Oxford University Hospitals National Health Service (NHS) Foundation Trust, Oxford, United Kingdom

**Keywords:** SARS-CoV-2, vaccine, COVID-19, adolescents, immunity

## Abstract

**Introduction:**

The key to understanding the COVID-19 correlates of protection is assessing vaccine-induced immunity in different demographic groups. Young people are at a lower risk of COVID-19 mortality, females are at a lower risk than males, and females often generate stronger immune responses to vaccination.

**Methods:**

We studied immune responses to two doses of BNT162b2 Pfizer COVID-19 vaccine in an adolescent cohort (n = 34, ages 12–16), an age group previously shown to elicit significantly greater immune responses to the same vaccine than young adults. Adolescents were studied with the aim of comparing their response to BNT162b2 to that of adults; and to assess the impacts of other factors such as sex, ongoing SARS–CoV–2 infection in schools, and prior exposure to endemic coronaviruses that circulate at high levels in young people. At the same time, we were able to evaluate immune responses to the co-administered live attenuated influenza vaccine. Blood samples from 34 adolescents taken before and after vaccination with COVID-19 and influenza vaccines were assayed for SARS–CoV–2-specific IgG and neutralising antibodies and cellular immunity specific for SARS–CoV–2 and endemic betacoronaviruses. The IgG targeting influenza lineages contained in the influenza vaccine were also assessed.

**Results:**

Robust neutralising responses were identified in previously infected adolescents after one dose, and two doses were required in infection-naïve adolescents. As previously demonstrated, total IgG responses to SARS–CoV-2 Spike were significantly higher among vaccinated adolescents than among adults (aged 32–52) who received the BNT162b2 vaccine (comparing infection-naïve, 49,696 vs. 33,339; p = 0.03; comparing SARS-CoV–2 previously infected, 743,691 vs. 269,985; p <0.0001) by the MSD v-plex assay. There was no evidence of a stronger vaccine-induced immunity in females compared than in males.

**Discussion:**

These findings may result from the introduction of novel mRNA vaccination platforms, generating patterns of immunity divergent from established trends and providing new insights into what might be protective following COVID-19 vaccination.

## Introduction

The BNT162b2 Pfizer-BioNTech COVID-19 vaccine was authorised for 12–15 year olds in June 2021 in the United Kingdom by the Medicines and Healthcare Products Regulatory Agency (1), with an initial 30 μg dose administered in the winter of 2021 and a second dose administered in early 2022 (2). In the United Kingdom, the first dose of BNT162b2 was administered to adolescents alongside the AstraZeneca intranasal seasonal live-attenuated influenza vaccine (LAIV) FluenzTetra, presenting a unique opportunity to study vaccine-induced immunity in this age group.

Older age is a primary risk factor for severe COVID-19, perhaps due to reduced immune capacity with age, driven by persistent inflammation and cellular dysfunction (3). The overall death rate of COVID-19 was 0.66%, increasing to 7.8% in the 80s (4). The majority of young people experience mild COVID-19; severe disease and multisystem inflammatory syndrome only occur in a minority of paediatric patients (5). Adolescents and children display rapid and adaptable immune responses that may contribute to improved resolution of infections, such as abundant IgM memory B cells, broad and rapidly produced natural antibodies, and lower inflammatory cytokine responses (6, 7). Differential COVID-19 outcomes between adults and children may also be influenced by pre-existing immune responses to endemic coronaviruses that circulate at higher levels in children (6). Notably, adolescents between 12 and 15 years of age generate 1.76-fold higher nAb responses to BNT162b2 than those aged 16–25 years, indicating either potential age-related changes in immune response even during adolescence or an increase in cross-reactivity with endemic coronaviruses that enhance vaccine responsiveness and decline with age (8). However, humoral responses to HCoVs have been associated with worse COVID-19 outcomes through the inhibition of novel responses to SARS-CoV-2 as a result of immune imprinting or ‘original antigenic sin’ (9). Children have been reported to display higher immunity to endemic HCoVs than adults (10), perhaps because of the high circulation of viruses in schools. Finally, older individuals are more likely to have immunodeficiencies or chronic diseases, which increases their risk of severe COVID-19.

In addition to age, understanding the role of sex in the vaccine response is crucial for the development of more effective vaccines. Adult females aged 18-49 have been shown to generate two-fold greater antibody responses to trivalent influenza vaccines (11), and adult females are more at risk for serious adverse events (SAEs) after vaccination, including after the ChAdOx1 Oxford-AstraZeneca COVID-19 vaccine (12–14). In one study, females administered a half-dose influenza vaccine produced marginally stronger antibody responses than age-matched males who received full-dose vaccine (11). Female children under five also display stronger antibody responses following vaccination against measles (15), diphtheria (16), and hepatitis A (17), although the literature on the subject is often variable, with some evidence of greater immune responses to vaccines, such as measles in males (18), or no significant difference between the sexes (19). Nevertheless, vaccine-induced immune responses in females could potentially facilitate reduced dosing regimens, which may minimise the incidence of SAEs, improve vaccine uptake, and improve vaccine supply. However, young males experience more vaccine-induced myocarditis after BNT162b2 treatment, suggesting that immune responses to mRNA vaccines may be differentially influenced by sex (20, 21). Adolescents undergoing puberty face significant changes in the levels of sex hormones such as testosterone and oestrogen, which are known to modulate immunity to SARS-CoV-2 and influenza (22, 23).

To explore sex- and age-specific differences in humoral and cellular immunity to BNT162b2, we studied adolescent and adult cohorts in the United Kingdom that received this vaccine. Data collected from adolescents in this study were compared to the Protective Immunity from T cells in the Healthcare Workers (PITCH) cohort of vaccinated healthcare workers (HCWs) aged 32–52 years, who received two doses of BNT162b2 and also represented a mixture of previously infected and infection-naïve individuals (24). We explored age-specific effects on immunity within the adolescent cohort as well as between adolescents and adults. Furthermore, we examined whether sex-specific immune effects were evident. As not all adolescents also received LAIV, we were also able to assess whether co-administration of LAIV appeared to influence the magnitude of the response to BNT162b2. Furthermore, many studies on adolescent responses to BNT162b2 have used prior SARS-CoV-2 infection as an exclusion criterion (8, 25). Here, we enrolled both SARS-CoV-2 infection-naïve and previously infected adolescents to understand the role of prior or ongoing infection in the vaccine response.

## Results

### Cohort description

**Figure 1:**
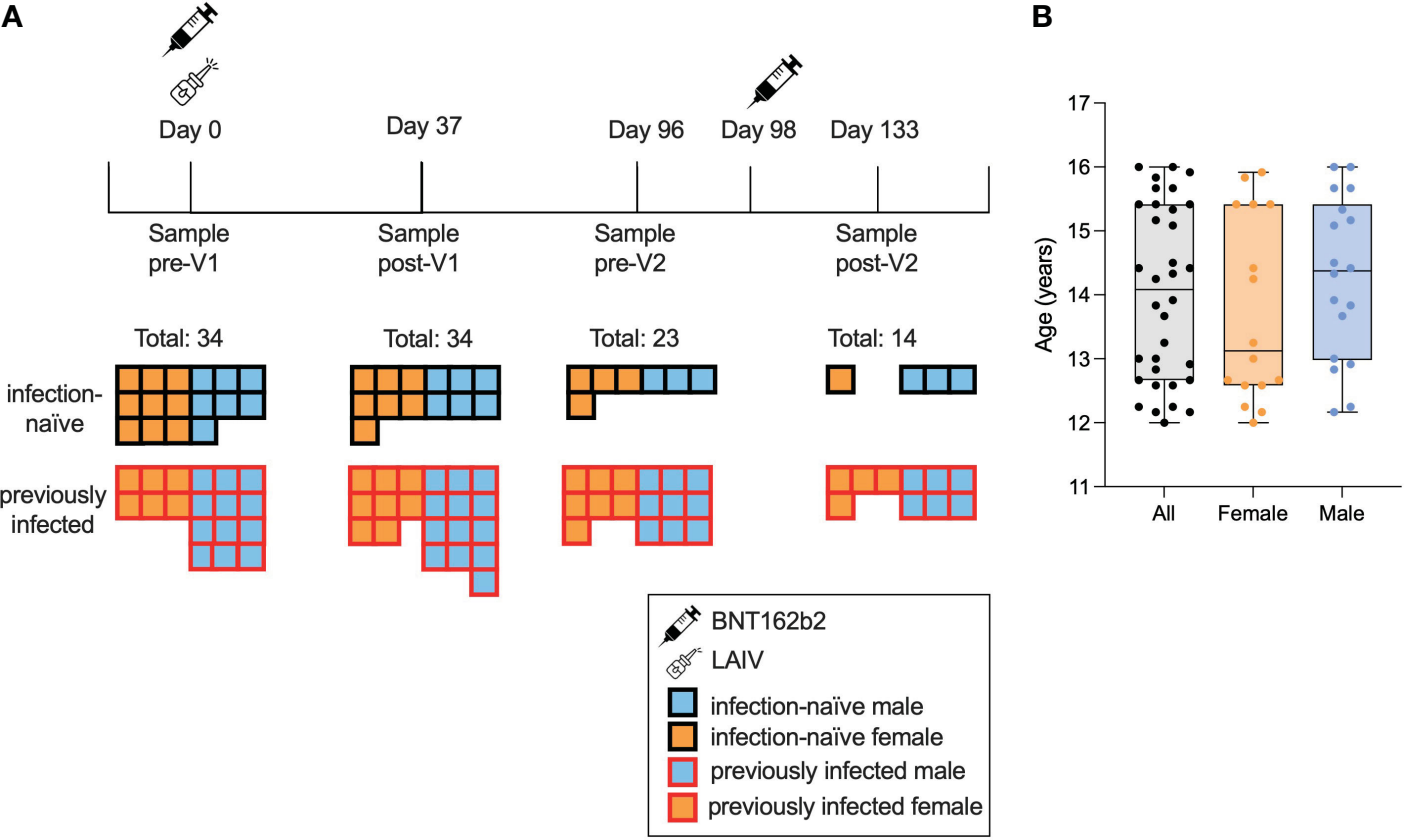
Characteristics of the study cohort. A total of 34 adolescents were enrolled and provided consent, of whom 18 were seropositive for S or N pre-Vx1. Samples were taken pre-Vx1 on the day of vaccination, a mean of 37 days post-Vx1, 2 days pre-Vx2, and 35 days post-VX2 **(A)**. The median age was 13 years 1 month for females (orange) and 14 years 5 months for males (blue) **(B)**.

In November and December 2021, 34 adolescents aged between 12 and 16 years were recruited into the study through their enrolment at schools in Oxford, UK (**Figure 1A**). All 34 individuals received the BNT162b2 vaccine; 26 (76%) received LAIV on the same day as the first dose of BNT162b2. Approximately 47% of the individuals (n = 16) were female, and the median age was 14.1 years (12.2–16) (**Figure 1B**). A total of 33 individuals were Caucasian and one was Asian. None of the individuals were taking any regular medication. All 34 individuals were sampled before the first dose (pre-Vx1) and after the first dose (post-Vx1), 23 individuals were sampled pre-Vx2 and 14 were sampled post-Vx2, giving a dropout rate of 41% over the course of the study.

The adult cohort to which adolescent data were compared was the PITCH cohort of vaccinated HCWs (24, 26). This cohort consisted of 589 adults aged 32–52 years who had received two doses of BNT162b2 28 days apart. IgG data from 79 adults and neutralising antibody (nAb) data from 10 adults were used for comparison with data from adolescents. These samples were randomly selected from the PITCH dataset to include roughly equal numbers of infection-naïve and previously infected samples. Only 10 individuals were included in the nAb data, as they were all available at the time.

### Humoral immune responses to BNT162b2 vaccination

**Figure 2:**
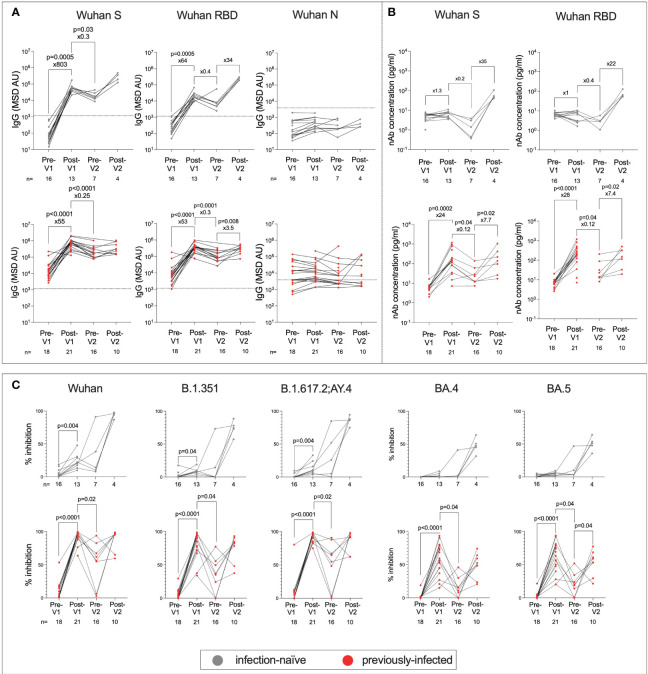
Humoral responses following first and second doses of BNT162b2 in previously infected and infection-naive adolescents. Anti-S, RBD and N IgG in infection-naive (grey) and previously infected (red) adolescents **(A)**. The thresholds for IgG positivity were obtained from previous studies (26). nAbs targeting S and RBD in infection-naïve (grey) and previously infected (red) adolescents using MSD ACE2-Spike binding inhibition assays **(B)**. Percent inhibition of SARS-CoV-2 S-ACE2 binding as measured by MSD ACE2 inhibition assay in infection-naive (grey) and previously infected (red) adolescents targeting common SARS-CoV-2 lineages: Wuhan, B.1.351(Beta), B.1.617.2/AY.4 (Delta), BA.4 and BA.5 (Omicron) **(C)**. P-values from Wilcoxon signed-rank tests. Fold-change refers to the difference between the total group medians.

To evaluate the immunogenicity of the BNT162b2 vaccine among adolescents, we first characterised humoral responses using MSD-platform immunoassays to quantitatively measure the total immunoglobulin G (IgG) response to the SARS-CoV-2 Spike (S), the receptor-binding domain (RBD) of S, and SARS-CoV-2-N (**Figure 2A**). Both infection-naïve and previously infected adolescents showed significantly greater IgG responses to S post-Vx1 than to pre-Vx1 (median: 61 vs. 49,696, ×803, p = 0.0005 and 13,409 vs. 788,568, ×55, p <0.0001, respectively, Wilcoxon signed-rank test) and greater anti-RBD IgG responses (263 vs. 16,861, ×64, p = 0.0005 and 6,556 vs. 351,068 ×53, p <0.0001, respectively, Wilcoxon signed-rank test) (**Figure 2A**) (**Supplementary Figures 1A, B**). Anti-S and RBD IgG responses increased post-Vx2 in all groups, but only anti-RBD IgG increased significantly and only in previously infected individuals (90,067 vs. 318,687, ×3.5, p = 0.008, Wilcoxon signed-rank test), although this is likely because only four infection-naïve individuals were included post-Vx2 due to the high drop-out rates over the course of the study. Notably, two doses of BNT162b2 in infected individuals gave similar levels of IgG to one dose of the vaccine in previously infected individuals. Recognising the multitude of comparisons made in this section and the risk of committing a type 1 error, a Bonferroni correction for multiple comparisons for the 12 tests conducted for S and RBD demonstrated that six associations remained significant at an alpha value of p = 0.004. These are the increased IgG responses to S and RBD post-Vx1 in both groups, and the waning of response between post-Vx1 and pre-Vx2 time points in previously infected individuals only.

Since nAbs as well as total IgG are reported to be a correlate of protective immunity against symptomatic COVID-19 (27), we next assessed a surrogate of nAb activity using the MSD-platform ACE2 inhibition assay (28), which is well correlated with live virus neutralisation assays (24, 26, 29, 30). In contrast to IgG responses, only previously-infected individuals generated increased nAb responses following the first dose of vaccine (6 vs. 149, ×24, p = 0.0002, Wilcoxon signed-rank test) (**Figure 2B**), and fold change in nAb response to S and RBD was higher in previously-infected individuals post-Vx1 compared to infection-naïve individuals (S: 24 vs. 1.3, p <0.0001 and RBD: 28 vs. 1, p = 0.0002, respectively, Mann–Whitney tests) (**Supplementary Figures 1C, D**). After two doses of BNT162b2, infection-naïve individuals reached nAb titres similar to those of previously infected individuals after one dose, supporting the idea that two doses of vaccine are required for a robust neutralising response in infection-naïve individuals. Again, to account for the six tests undertaken under this hypothesis, the Bonferroni correction with an alpha value of p=0.008 revealed that only post-Vx1 nAb responses in previously infected individuals remained significantly increased. The reduction in nAb response pre-Vx2 to levels lower than pre-Vx1 is surprising and may be a result of batch effects in the analysis. Alternatively, higher nAb levels pre-Vx1 may be a result of cross-reactivity with endemic HCoVs following a recent infection.

To determine how the breadth of the nAb response to SARS-CoV-2 variants is impacted by vaccination and prior infection, the MSD-platform ACE2 inhibition assay was carried out against the common variants of SARS-CoV-2 in both infected and previously infected individuals (**Figure 2C**; **Supplementary Figure 2**). Notably, previously infected individuals showed broad nAb responses against all the studied variants following the first dose, whereas high-titre nAb responses against these variants were only observed following the second dose in infection-naive individuals. Using Bonferroni correction with an alpha value of p = 0.002, for the 30 comparisons made under this hypothesis, % inhibition of ACE2-S binding remained significantly elevated post-Vx1 in previously infected individuals only. In previously infected individuals, median responses were highest post-Vx1 and post-Vx2 for the Wuhan, B.1.351, and B.1.617.2;AY.4 strains, whereas responses to BA.4 and BA.5 were more varied. This may be because some adolescents were infected with the former strains before the study was carried out, whilst some adolescents who became infected during the study were likely infected with BA.4 and BA.5. Therefore, a range of nAb responses to BA.4 and BA.5 were expected in this group. In contrast, in the infection-naïve cohort, all individuals showed weaker responses to BA.4, BA.5 post-Vx2, and post-Vx2 than they do to the first three VOCs. This is likely because none of these individuals were infected with BA.4 and BA.5.

### Cellular immune responses to BNT162b2 vaccination

**Figure 3:**
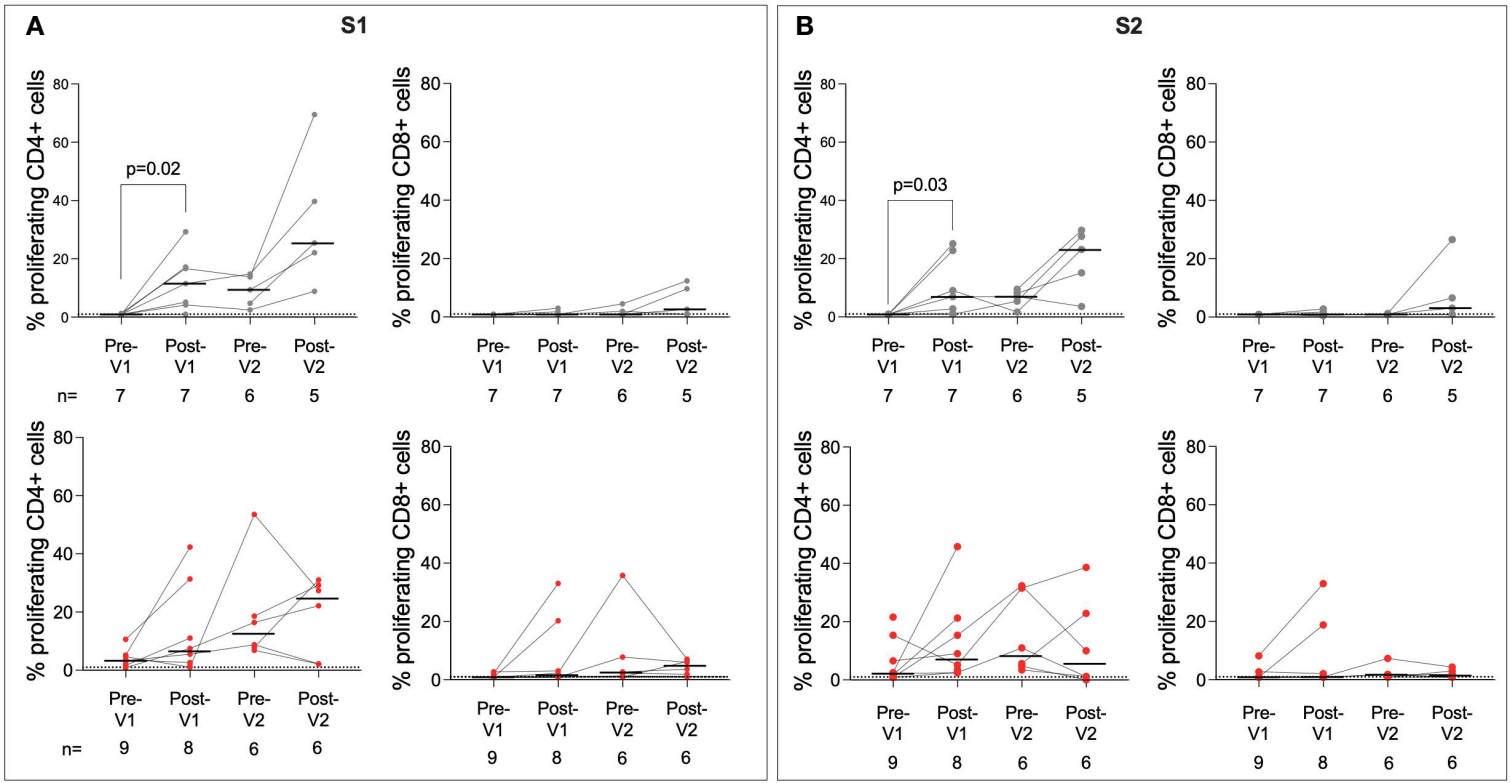
T-cell responses to SARS-CoV-2 S are boosted post-Vx1 and post-Vx2. CellTrace Violet stains were used to assess proliferating CD4+ and CD8+ T cells targeting the S1 region of S **(A)** and the S2 region of S **(B)** in infected (grey) and previously infected (red) individuals. Data show proliferating cells as a percentage of the parent populations with subtracted DMSO background values. Thresholds for positivity were set at 1, as determined in previous studies (31). P-values from Wilcoxon signed-rank tests. Fold-change refers to the difference between the total group medians. Values below 1% were given nominal values of 0.9%.

Next, we characterised the cellular immune response in adolescents following the first and second doses of BNT162b2 using a CellTrace Violet (CTV) proliferation assay (**Figure 3**; **Supplementary Figures 3**, **4**; **Supplementary Table 1**). The proliferation assay was chosen as it has been used previously to quantify SARS-CoV-2 specific T cells and has been shown to be sensitive to low-magnitude responses, perhaps due to the long incubation period (31). Infection-naïve individuals showed significantly increased responses to S1 post-Vx1 (p = 0.02, Wilcoxon test). However, the small number of individuals included in this analysis makes the interpretation difficult. In contrast to humoral responses to BNT162b2, SARS-CoV-2-specific CD4+ T-cell proliferative responses were similar in infection-naïve individuals compared to previously infected individuals after a single dose of vaccine. The T-cell response in previously infected individuals also increased following one dose of the vaccine, albeit not significantly. For CD4+ responses to S1 in particular, there was a general trend of an increased magnitude of response following vaccination. CD8+ responses were of lower magnitude, and no clear trend of increasing immunity following vaccination could be observed, although responses did increase following Vx1 in some individuals.

Although T-cell responses to HCoV-OC43 S2 and HCoV-HKU1 S2 were identified in several individuals, particularly in previously infected individuals, no significant impact of BNT162b2 vaccination on the magnitude of T-cell responses was observed. Responses appeared to peak pre-Vx2, which may be the result of batch effects within the assay or a delayed response following Vx1. Responses to M and N were also measured (**Supplementary Figure 4**); these did not increase significantly post-Vx1 or Vx2 treatment, as expected. Some individuals showed increased responses to M and N over the course of the study. As only S is included in BNT162b2, this may reflect reinfection with SARS-CoV-2 in the case of the previously infected group or infection with endemic HCoVs in the infection-naïve group.

### Higher magnitude antibody responses to BNT162b2 in adolescents versus adults

**Figure 4:**
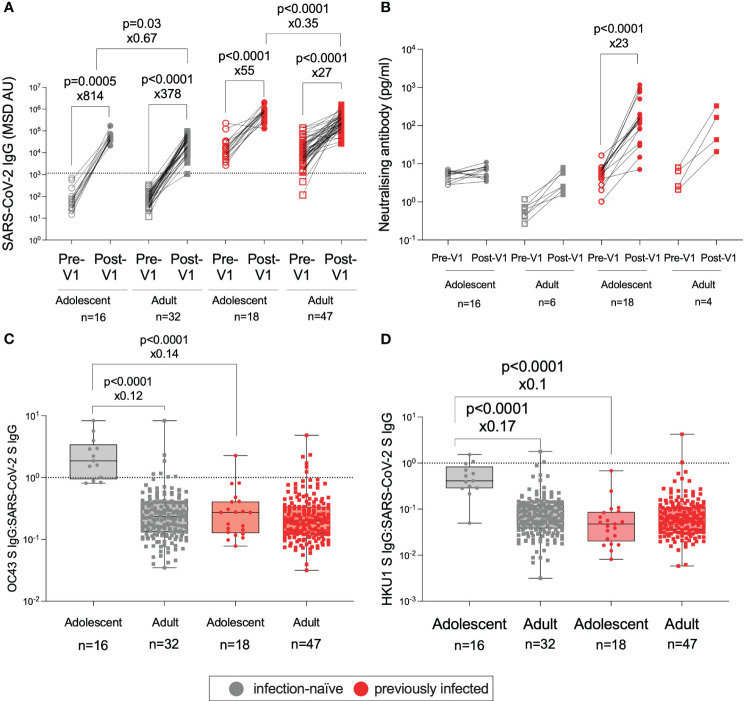
Age-specific effects on the humoral response to BNT162b2. IgG targeting S in infection-naïve adolescents (grey circles), infection-naïve adults (32–52 years) (grey squares), previously infected adolescents (red circles), and previously infected adults (red squares), pre-Vx1 (unfilled shapes) and post-Vx1 (filled shapes) as measured by an MSD v-plex immunoassay **(A)**. nAb concentration targeting S in infection-naïve and previously infected adolescents and adults as measured by an MSD ACE2-Spike binding immunoassay **(B)**. The ratio of IgG targeting HCoV-OC43 S to SARS-CoV-2 S **(C)** and the ratio of IgG targeting HCoV-HKU1 S to SARS-CoV-2 S **(D)** in infection-naive adolescents (grey circles), infection-naive adults (grey squares), previously infected adolescents (red circles), and previously infected adults (red squares) post-Vx1. P-values represent Mann–Whitney test values for unpaired data and Wilcoxon signed-rank test values for paired data. The fold change was calculated as the ratio of population medians.

The role of age in the immune response to vaccination was of particular interest in this study. To determine whether the responses observed in adolescents to the BNT162b2 vaccine were stronger than those observed in adults, as previously shown (8), we compared the adolescent data to humoral responses in adults (32–52 years) from the PITCH cohort 28 days after the first dose of BNT162b2 (**Figure 4**) (26, 32). PITCH is a consortium of universities and the UK Health Security Agency (UK HSA) with the aim of characterising infection-acquired and vaccine-induced immunity to SARS-CoV-2 in HCWs. Here, as reported for adolescents receiving two vaccines (8, 25), post-Vx1, infection-naive adolescents generated higher magnitude anti-S IgG responses than infection-naive adults (49,696 vs. 33,339, ×1.5, p = 0.03, Mann–Whitney test) and previously infected adolescents generated greater anti-S IgG responses than previously-infected adults (743,691 vs. 269,985, ×2.9, p <0.0001, Mann–Whitney test) (**Figure 4A**). Post-Vx1 nAb responses did not differ significantly between adolescents and adults, although the small number of previously infected adults (n = 4) included in this analysis limited this conclusion (**Figure 4B**). Infection-naïve adolescents appeared to have higher levels of pre-Vx1 nAbs than adults; the reasons behind this are unclear but may related to pre-existing cross-reactive immunity to endemic HCoVs. Alternatively, this may be an artefact of the noise at the lower limit of detection of the assay. Despite this higher baseline, nAb titres did not change significantly post-Vx1 in the infection-naïve adolescents. To account for the six comparisons undertaken under this hypothesis and to reduce the possibility of committing a type 1 error, Bonferroni correction was employed. With a new alpha value of 0.008, all but one comparison (infection-naïve adults vs. adolescents) remained significant. It is also possible that the difference in group size (n = 34 adolescents, n = 79 adults) influenced the results of this analysis.

To investigate why infection-naive adolescents did not generate significantly increased nAb responses post-Vx1 despite a strong total IgG response, we sought to address the hypothesis that cross-reactive antibody responses to endemic HCoVs might be present at higher levels in infection-naïve adolescents, thereby interfering with the generation of novel SARS-CoV-2-specific responses to BNT162b2, as has been suggested previously (9, 33).

Our data supported the hypothesis that cross-reactive antibody responses to HCoVs are associated with weaker vaccine-induced neutralising responses: in this study, the ratio of IgG targeting betacoronaviruses HCoV-OC43 and HCoV-HKU1 S to IgG targeting SARS-CoV-2 S was significantly higher in infection-naive adolescents versus infection-naive adults (1.9 vs. 0.2, ×8.3, p <0.0001; 0.4 vs. 0.06, ×5.9, p <0.0001, Mann–Whitney tests) and versus previously-infected adolescents (1.9 vs. 0.3, ×7.1, p <0.0001; 0.4 vs. 0.04, ×10, p <0.0001, Mann–Whitney test) post-Vx1 (**Figures 4C, D**). There was no significant difference in the ratio between infection-naïve adolescents and previously infected adults, perhaps due to several previously-infected adults had a high HCoV:SARS-CoV-2 IgG ratio. Furthermore, the ratio of IgG targeting HCoV-OC43 and HCoV-HKU1 S to IgG targeting SARS-CoV-2 S was significantly negatively correlated with the nAb response (OC43: r = −0.84, p<0.0001; HKU1: r= − 0.75, p <0.0001) in all adolescents, although this significance was lost when adolescents were divided into infection-naïve and previously infected groups (**Supplementary Figure 5**). Again, considering the eight comparisons made for this hypothesis, utilising the Bonferroni correction revealed a new alpha value of p = 0.006. All four significant differences remained significant after correction.

### Sex differences in response to BNT162b2 vaccination

**Figure 5:**
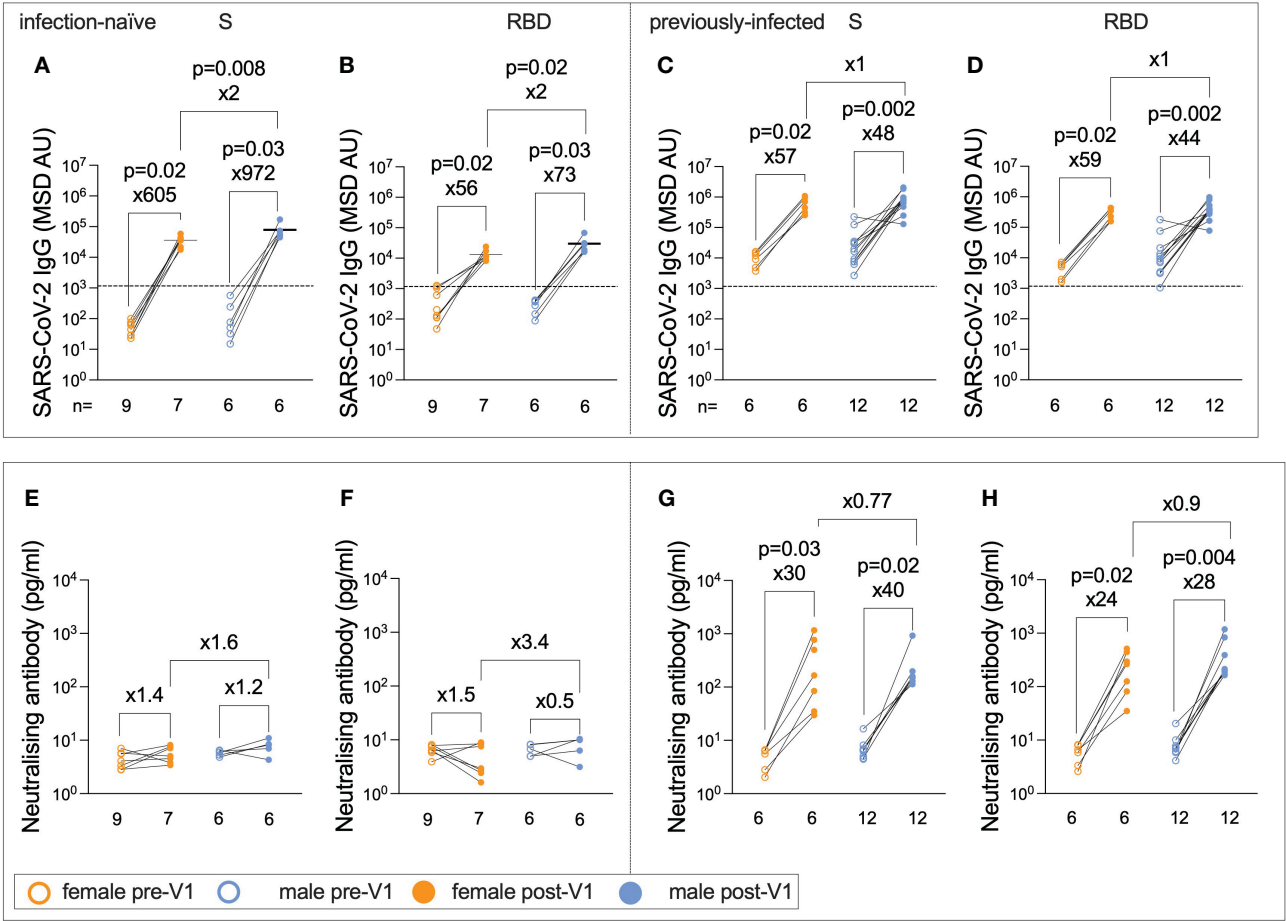
Infection-naïve male adolescents generate greater post-Vx1 IgG responses than do female adolescents. IgG targeting S **(A)** and RBD **(B)** in infection-naive adolescents pre-Vx1 (unfilled circles) and post-Vx1 (filled circles) in females (orange circles) and males (blue circles), as measured using an MSD v-plex immunoassay. IgG targeting S **(C)** and RBD **(D)** in previously infected adolescents pre-Vx1 and post-Vx1 in females and males. Concentration of nAbs targeting S **(E)** and RBD **(F)** in infection-naive adolescents measured by an MSD ACE2-Spike binding inhibition assay. Concentration of nAbs targeting the S **(G)** and RBD **(H)** in previously infected adolescents. P-values represent Wilcoxon test values for paired data and Mann–Whitney test values for unpaired data. The fold change was calculated as the ratio of population medians.

Females typically elicit stronger IgG responses than males following vaccination (12, 13, 23, 34), including after influenza vaccines (11, 23). Surprisingly, infection-naïve males generated significantly higher post-Vx1 IgG targeting both SARS-CoV-2 S and RBD than females (62,270 vs. 36,951, ×2, p = 0.008; 23,860 vs. 11,443, ×2, p = 0.02, respectively; Mann–Whitney tests) (**Figures 5A, B**). There was no significant difference in IgG response between the sexes of previously infected individuals (**Figures 5C, D**). Furthermore, there was a trend towards a stronger RBD and S nAb response post-Vx1 in infection-naive males compared to infection-naive females, although this was not significant (p = 0.07 and p = 0.15, respectively, Mann–Whitney tests) (**Figures 5E, F**), and there was no significant difference in nAb response between previously infected males and females (**Figures 5G, H**). There was no significant difference in baseline IgG responses between males and females. Furthermore, using a Bonferroni correction to consider the 24 comparisons made under this hypothesis, no comparisons remained significant with a new alpha value of p = 0.002. In addition, the small number of individuals in each group likely affects the statistical analysis undertaken. Furthermore, there was no significant difference in IgG-targeting endemic HCoVs between males and females in this cohort, which may have been a potential confounder in this analysis. There were no reported sex differences in the humoral response to BNT162b2 in adults in previous studies (24, 26).

### Humoral responses to LAIV administration

**Figure 6:**
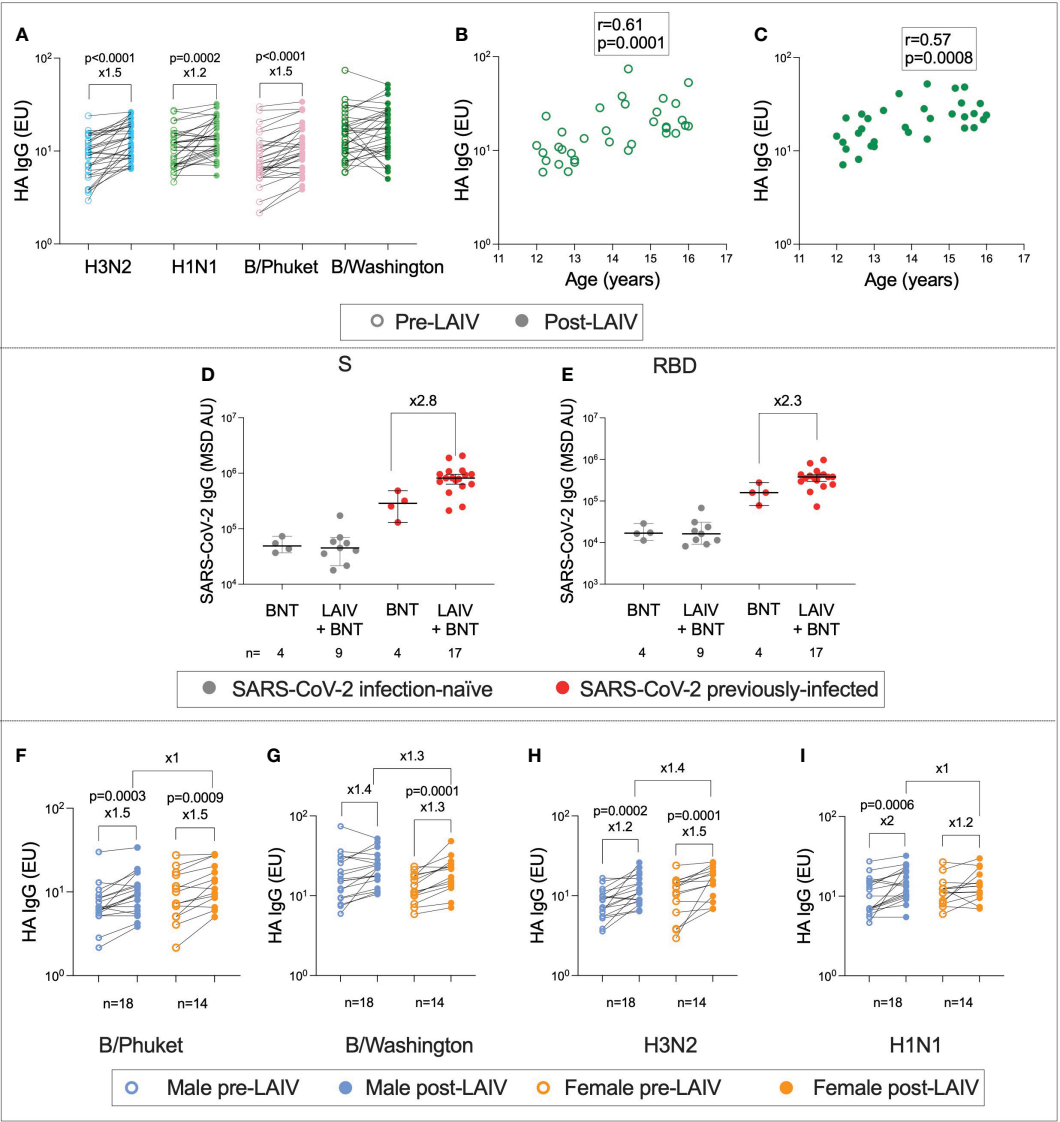
Age- and sex-specific immunity to influenza following LAIV administration. IgG targeting haemagglutinin (HA) pre- (unfilled circles) and post- (filled circles) LAIV administration for the four influenza lineages **(A)** (P-values from Wilcoxon tests). Correlation between age and IgG targeting HA for the B/Washington lineage pre-Vx1 **(B)** and post-Vx1 **(C)** (Spearman rank r- and p-values). IgG targeting SARS-CoV-2 S **(D)** and RBD **(E)** in infection-naïve (grey) and previously infected (red) adolescents who received the BNT162b2 vaccine alone (BNT) or co-administered with the LAIV (LAIV + BNT) (Mann–Whitney p-values). IgG targeting HA pre- (unfilled circles) and post- (filled circles) LAIV administration in males (blue) and females (orange) for the four influenza lineages **(F–I)** (Wilcoxon p-values).

In addition to the immune response to BNT162b2, the co-administration of LAIV enabled the characterisation of immunity against influenza following vaccination. To determine the effect of LAIV on lineage-specific anti-haemagglutinin (HA) IgG titres, enzyme-linked immunosorbent assays (ELISAs) were performed on pre- and post-Vx1 samples from the 26 individuals who received LAIV (**Figure 6**). As expected, IgG titres were significantly higher post-Vx1 for A/Cambodia (H3N2), A/Victoria (H1N1), and B/Phuket (Yamagata) (9.3 vs. 13.9, ×1.5, p <0.0001; 11 vs. 13.4, ×1.2, p = 0.0002; 7 vs. 10.2, ×1.5, p <0.0001; respectively, Wilcoxon signed-rank tests) (**Figure 6A**). Employing a Bonferroni correction to account for the four comparisons under this hypothesis resulted in an alpha value of 0.01. All three differences remained significant at this alpha value. Surprisingly, post-Vx1 anti-HA IgG responses towards the B/Washington (Victoria) lineage were not significantly increased compared to pre-Vx1. A possible explanation is that responses to B/Washington (Victoria) were strongly correlated with age at both pre- and post-vaccine time points (r = 0.61, p = 0.0001, r = 0.57, p = 0.0008, respectively, Spearman rank test) (**Figures 6B, C**). In contrast, there was no correlation between age and post-Vx1 IgG for A/Cambodia (H3N2) or B/Phuket (Yamagata), and for A/Victoria (H1N1), pre-Vx1 IgG levels only weakly correlated with age (r = 0.39, p = 0.02, Spearman rank test). This pattern suggests that natural exposure to B/Washington (Victoria) is so frequent in this cohort that vaccination against this strain of influenza does not significantly add to the natural immunity that accumulates during adolescence. Pre-existing immunity to influenza has been widely described from prior infection and vaccination, in support of this finding (35, 36).

Unexpectedly, and in contrast to previous studies (37), adolescents previously infected with SARS-CoV-2 who received both BNT162b2 and LAIV appeared to generate over two-fold-higher higher post-Vx1 IgG targeting both S and RBD compared to adolescents previously infected with SARS-CoV-2 who received BNT162b2 alone. However, as this analysis involved a very small number of individuals, the statistical analysis was not appropriate (**Figures 6D, E**). Additionally, using Bonferroni correction to account for multiple testing, with a new alpha value of 0.012, these differences were no longer significant. In terms of demographics, the four BNT-alone infection-naïve individuals were two males and two females, aged 12 years 6 months to 16. The nine BNT+LAIV infection-naïve individuals included four males and five females, aged 12 years 3 months to 15 years 11 months. The four BNT-alone previously infected individuals were two males and two females, aged 13 years 8 months to 14 years 5 months. The 17 BNT + LAIV previously infected individuals were six females and 11 males aged 12–16 years. We did not find a sex difference in the IgG response to LAIV (**Figures 6F–I**).

To assess whether individuals who generated strong vaccine responses to BNT162b2 also generated higher magnitude LAIV responses, the correlation between anti-S IgG post-Vx1 and anti-HA IgG targeting the four influenza lineages was calculated. There was no correlation between anti-S IgG and anti-HA IgG in B/Phuket, B/Washington, or A/Cambodia (H3N2) (B/Phuket: r = −0.1, p = 0.6; B/Washington: r = 0.1, p = 0.62; A/Cambodia (H3N2): r = 0.18, p = 0.37), although there was a moderate negative correlation with A/Victoria (H1N1) (r = −0.43, p = 0.03). However, to correct for multiple comparisons using the Bonferroni correction, with a new alpha value of 0.01, this was no longer significant.

## Discussion

Understanding the quantitative markers of vaccine immunogenicity, as well as confounding patient demographic factors, will help to better define the correlates of protection against SARS-CoV-2 and improve the interpretability of future vaccine trials. In this study, by assessing humoral and cellular immunity to SARS-CoV-2, influenza virus, and endemic HCoVs in adolescents receiving BNT162b2, we identified several intriguing patterns that shed light on the immunogenicity of BNT162b2 in this age group. These include the role of prior SARS-CoV-2 infection in promoting a quicker and more neutralising vaccine response, the appearance of stronger humoral responses in adolescents than in adults, and a lack of sex difference following both BNT162b2 and the LAIV.

Due to the discrepancy between IgG and nAb responses in infection-naive adolescents, these data support the use of nAb titre as well as total IgG when assessing vaccine immunogenicity (38, 39). Other studies have established that BNT162b2 and CoronaVac inactivated virus vaccine elicit robust nAb responses post-Vx2 in infection-naive adolescents (8, 40). The totality of the data described herein suggests that a robust nAb response is prompted in infection-naive adolescents after two doses, but previously infected adolescents only require one dose. Previous studies in adults have differed in their evaluation of vaccine-induced versus infection-induced humoral immunity, but these data show that, at least in adolescents, a similar IgG response is elicited after natural infection and one vaccine dose compared to two vaccine doses alone (41). The longevity of these responses is uncertain due to the lack of an extended follow-up in this cohort but should be the focus of future studies.

Other research has shown that two doses of BNT162b2 elicit robust T_H_1 T-cell responses in adults, with widespread interferon-gamma (IFNγ) production (26, 42). S-specific T-cell responses following vaccination with BNT162b2 were generated post-Vx2, but not post-Vx1 in another cohort of infection-naive adolescents (25). This contrasts with the data described herein, where one dose of BNT162b2 was sufficient to induce an increase in S-specific CD4+ T cells in infection-naive adolescents. Similar to the IgG response, SARS-CoV-2-specific T-cell responses post-Vx1 in previously infected individuals reached similar frequencies to post-Vx2 in infection-naive individuals. However, further studies on cellular immunity following BNT162b2 are required to supplement the small number of individuals studied here.

BNT162b2 has been shown to promote greater IgG production in adolescents compared to adults post-Vx1 (8, 25). Similarly, both infection-naive and previously infected adolescents generated stronger IgG responses than adults, although this only remained significant in previously infected adolescents when correcting for multiple comparisons. However, only previously infected adolescents generated a strong and broad nAb response targeting multiple variants, and infection-naïve adolescents appeared to generate more cross-reactive antibodies following their first dose of BNT162b2 compared to both infection-naïve adults and previously infected adolescents, as indicated by the higher HCoV:SARS-CoV-2 IgG ratios in infection-naïve adolescents. One interpretation for these patterns is immune imprinting, wherein prior exposure to circulating endemic coronaviruses negatively affects vaccine-induced immunity. Higher levels of cross-reactive IgG have been described in children than in adults (10, 43, 44), which may result in a stronger memory B-cell response, although with no significant boosting of nAb titres, following the first dose of BNT162b2 in infection-naive adolescents. In previously infected adolescents, exposure to SARS-CoV-2 may overcome immune imprinting and enable a robust nAb response. This is supported by the strong negative correlation between the HCoV:SARS-CoV-2 IgG ratio and nAb response.

Immune responses to many adult and childhood vaccines, as well as responses to natural infection with viral pathogens, are consistently higher in females and associated with increased inflammation and autoimmunity as well as CD4+-skewed T-cell responses and greater B cell activation and IgG production (12, 13, 23, 34). Female IgG responses to influenza vaccines, such as the trivalent inactivated influenza vaccine, have been shown to be twice the magnitude of male IgG responses, and females also report more frequent SAEs to viral vaccines (13, 34). One exception to this trend is COVID-19 mRNA vaccines, for which vaccine-induced myocarditis is more frequent in young males (20, 21, 45). Lower peak anti-S IgG levels were identified in males following two doses of BNT162b2 (46). However, although geometric mean nAb titres to BNT162b2 were slightly higher in females following two doses of BNT162b2 in 12–15- and 16–25-year-olds, this difference was not significant in the US Food and Drug Administration open-label extension report for BNT162b2 (47). Notably, in this cohort, we observed increased post-Vx1 anti-S and anti-RBD IgG responses in infection-naive males compared to infection-naive females, in contrast to expectations based on other vaccines such as inactivated influenza vaccines (11, 13). However, this significance was lost when multiple comparisons were corrected. In addition, no sex differences in immune response have been reported for the adult cohort in previous studies (24, 26). We did not observe a significant sex difference in anti-HA IgG titres following LAIV, which is surprising in the context of established literature (12, 13) but may be obscured by the very small increase in anti-HA IgG post-Vx1 in this cohort, the effect of a live-attenuated rather than inactivated influenza vaccine, the use of different serological assays, or the result of co-administration with BNT162b2 (48). Furthermore, there was no sex difference in anti-SARS-CoV-2 IgG levels for either infected or previously infected adults from the PITCH dataset.

Finally, the correlation between B/Washington influenza IgG responses with age in 12–16-year-olds, as well as the lack of anti-B/Washington HA IgG boosting following LAIV, suggests recent exposure to the B/Washington strain of influenza in this cohort. Our findings that co-administration of BNT162b2 with the LAIV improves IgG response in previously-infected individuals is in contrast with findings for NV×-COV12373, where co-administration with inactivated quadrivalent influenza vaccines reduced SARS-CoV-2-specific IgG titres (37). However, studies of the co-administration of COVID-19 mRNA vaccines with quadrivalent influenza vaccines in adults have reported no reduction in antibody response compared to the administration of mRNA vaccines alone (49, 50). A potential explanation for the improved anti-S IgG responses following co-administration may be the increased innate immune activation due to intranasal LAIV administration, particularly in the nasal mucosa, leading to greater SARS-CoV-2-specific local T-helper cell activation. However, analysis with a greater number of adolescents receiving either BNT162b2 alone or BNT162b2 alongside the LAIV is required to make further conclusions on this hypothesis.

This study had several limitations. The small number of adolescents assayed in this cohort makes broad conclusions difficult, particularly when making comparisons between small subgroups such as co-administered LAIV/BNT162b2 and BNT162b2-alone individuals. No mucosal samples were collected, and mucosal immunity was not assessed in this cohort. Neutralisation responses to SARS-CoV-2 were estimated using an MSD-ACE2 inhibition assay. This has been shown to correlate with live virus assays (24, 26, 29), but live virus neutralisation is likely to be a more accurate measure of nAbs. In addition, no neutralisation assays were performed for influenza lineages, which would have shed further light on the functionality of humoral immunity against influenza. Owing to cell availability, T-cell proliferation assays were only performed on a limited number of individuals in the cohort, reducing confidence in the generalisability of the results. The adolescent cohort was compared with the PITCH cohort of adults. Although this cohort was also divided into infection-naïve and previously infected individuals and received the same vaccine, there are other potential confounders that limit this comparison, such as the sampling of adults 28 days post-vaccination rather than 35 days, as well as comorbidities that may be more prevalent in adults versus adolescents. Furthermore, the lack of an extended follow-up in this study makes assessments of immune durability impossible but should be the focus of future studies.

Another potential confounder for this study is that adolescents of this age group are likely to be at different stages of puberty and therefore have diverse levels of testosterone, oestrogen, and progesterone. Furthermore, males experience puberty at older ages than females; therefore, the sex difference identified herein may result from the confounding effects of puberty. If many male adolescents did not go through puberty at the time of sampling, the increased humoral responses to vaccination in males may result from the absence of immunosuppressive effects of androgens. To ensure that males in this cohort had entered puberty, steroid hormones, including testosterone, dihydrotestosterone (DHT), and progesterone, were measured by tandem mass spectrometry; these data are the focus of a future publication. All but the two youngest males (12 years, 2 months and 12 years, 10 months) demonstrated pubertal androgen levels. Testosterone levels correlated with age in males only (r = 0.47, p = 0.05). This promotes confidence in the results of the comparisons between sexes, as most males had undergone puberty at the time of sampling. In addition, in this study, the median age of males was approximately one year older than females, which may have affected the results of the study.

Taken together, these data paint a complex picture of vaccine-induced immunity in adolescents, with a potential role for sex and age differences in determining antibody responses to vaccination. These findings have important implications for paediatric vaccination regimens, such as the potential benefit of co-administration with influenza vaccines, and the necessity to consider sex and age when studying vaccine-induced immunity.

## Materials and methods

### Ethics

This longitudinal cohort study was conducted between November 2021 and February 2022. Eligible participants were healthy adolescents aged 12–16 years who either had no history of SARS-CoV-2 infection or had experienced mild disease prior to enrolment. Eligible participants were identified by their participation in school-based vaccination. Written informed consent was obtained from all patients and ethical approval was obtained from the Central University Research Ethics Committee (reference: CUREC R71346/RE001). Healthy HCWs aged 32–52 years were recruited as part of the PITCH consortium of HCWs under the GI Biobank Study 16/YH/0247, approved by the Research Ethics Committee (REC) of Yorkshire and The Humber—Sheffield Research Ethics Committee on 29 July 2016, which was amended for this purpose on 8 June 2020.

### Sample collection and processing

For the BNT162b2 vaccination (dose 1 (Vx1) and dose 2 (Vx2)), patients received 30 μg of the vaccine intramuscularly. LAIV was administered immediately after Vx1 only; patients received 0.1mL intranasally in both nostrils. Whole blood samples from all 34 individuals were collected immediately before Vx1 (sample pre-Vx1). Samples from all 34 individuals were taken at a mean of 37 days after Vx1 (33-39]) (sample post-Vx1), from 23 individuals 2 days before Vx2 (0–8) and 96 days after Vx1 (81-114) (sample pre-Vx2), and from 14 individuals 35 days after Vx2 (30–40) (sample post-Vx2). All whole blood samples were processed on the same day as collection, as described in the *Materials and methods* section. All serum samples were tested for anti-Spike (S) and anti-nucleocapsid (N) IgG and were classified as seropositive if their anti-N IgG titre was above the previously determined MSD immunoassay cut-off at any point in the study or if their anti-spike (S) IgG titre was above the cut-off pre-Vx1 (26). Only individuals who became infected were included in the seropositive group at the time of seropositivity. The percentage of seropositive patients increased from 52% (n = 18 of 34) to (10 of 14) over the course of the study.

Whole blood samples were transported from the collection site to an academic laboratory and were processed on the same day. PBMCs and plasma were isolated as previously described (31). Briefly, PBMCs were isolated using Lymphoprep (1.077 g/ml, Stem Cell Technologies) via density gradient centrifugation. Plasma and PBMCs were collected, and plasma was centrifuged at 2,000×*g* for 10 min to remove platelets. PBMCs were washed twice with RPMI 1640 (Sigma) containing 10% heat-inactivated foetal calf serum, 2 mM L-glutamine and 1 mM penicillin/streptomycin (Sigma). An estimated 10 million cells were resuspended in the media and counted using a Muse Cell Analyser (Luminex Corporation, USA). Plasma and PBMCs were frozen and stored at −80°C for later use. Steroid hormone concentrations were quantified using tandem mass spectrometry by collaborators at the Imperial College London.

### MSD serological assays

IgG responses to SARS-CoV-2 S, N and RBD as well as the S proteins of HCoV-OC43, HCoV-NL63, HCoV-229E, HCoV-HKU1, SARS-CoV-1 and MERS-CoV were measured using a Meso Scale Diagnostics (MSD) V-plex immunoassay ‘Coronavirus panel 3’ (MSD, USA) according to the manufacturer’s protocol. The plates were incubated in Blocker A solution for 30 min at room temperature (RT) with shaking at 700 rpm. Plasma or serum was diluted 1:1,000 and 1:10,000 in Diluent 100, and a seven-point standard curve of the MSD reference standard beginning at 1:10 was prepared in duplicate. Three internal controls and an in-house control of convalescent serum were used, with Diluent 100 used as a blank. Plates were washed three times with MSD Wash Buffer, and samples and standards were added to the plate before incubation at RT for 2 h with shaking at 700 rpm. The plates were washed three times with MSD Wash Buffer, and the detection antibody solution was added. The plates were then incubated for 1 h at RT with shaking at 700 rpm. Plates were washed three times with MSD Wash Buffer. Neat MSD Gold Read Buffer was added, and the plates were read immediately on a MESO QuickPlex SQ 120 (MSD, USA). Data were analysed using MSD Discovery Workbench software. Thresholds for seropositivity were taken from analyses of pre-pandemic sera, as published elsewhere (26), and were defined as 1,160 AU/ml for SARS-CoV-2 S, 1,169 for RBD, and 3,874 for N.

nAb titres were quantified using Meso Scale Diagnostics ACE2 inhibition assays, ‘Panel 27,’ (analytes: SARS-CoV-2 S, SARS-CoV-2 S (B.1.351), SARS-CoV-2 S (B.1.617.2; AY.4), SARS-CoV-2 S (BA.2), SARS-CoV-2 S (BA.2.12.1), SARS-CoV-2 S (BA.2+L452M), SARS-CoV-2 S (BA.2+L452R), SARS-CoV-2 S (BA.3), SARS-CoV-2 S (BA.4), SARS-CoV-2 S (BA.5)) according to the manufacturer’s instructions. The plates were incubated in Blocker A solution for 30 min at RT with shaking at 700 rpm. Serum was diluted at 1:10 and 1:100, and a seven-point standard curve of MSD calibration reagent was prepared with 4-fold serial dilutions. Plates were washed three times with MSD Wash Buffer, and samples and calibrators were added to the plate. The plates were incubated at RT for 1 h, with shaking at 700 rpm. Sulfo-tagged ACE2 protein was added to the plate and incubated at RT for 1 h with shaking at 700 rpm. The plates were washed three times with MSD Wash Buffer and MSD Gold Read Buffer was added. The plates were read immediately on a MESO QuickPlex SQ 120 (MSD, USA). Data were analysed using MSD Discovery Workbench software. Results for VOCs were reported as % inhibition rather than pg/ml, as the standard included in the assay is specific for the Wuhan strain of SARS-CoV-2 only.

### Influenza ELISA assay

IgG responses to influenza A/Victoria (H1N1), B/Washington (Victoria), A/Cambodia (H3N2), and B/Phuket (Yamagata) HA antigens were measured using an indirect ELISA. HA antigens (The Native Antigen Company, Oxford) were diluted to 1 ug/ml in PBS and used to coat 535 Nunc-Immuno 96-well plates (Thermo Fisher Scientific, USA) overnight at 4°C (A/Victoria/2570/2019 (H1N1)pdm09-like virus (NCBI Accession Number: EPI1799581), amino acids 1–528 and C-terminal His-tag; Cambodia/e0826360/2020 (H3N2)-like virus (NCBI Accession Number: EPI1799580), amino acids 46–469 and C-terminal His-tag; B/Washington/02/2019 (B/Victoria lineage)-like virus (NCBI Accession Number: EPI1846769), amino acids 31–469 and C-terminal His-tag, B/Phuket/3073/2013 (B/Yamagata Lineage)-Like virus] (NCBI Accession Number: EPI1799823), amino acids 44–466 and C-terminal His-tag) Plates were washed three times in 0.1% PBS-Tween, before blocking with Casein-PBS Buffer for 1 h at RT. Plasma was diluted 1:200 in Casein-PBS Buffer and added to plates in duplicate. A ten-point standard curve of pooled highly reactive sera, beginning at 1:25, was prepared in duplicate and added to the plates. Casein-PBS Buffer was used as a negative control. The plates were incubated for 2 h at RT and washed six times with 0.1% PBS-Tween. The secondary antibody–goat anti-human IgG conjugated to alkaline phosphatase (Sigma, USA) was diluted 1:1,000 in Casein-PBS Buffer and added to the plates. The plates were incubated for 1 h at RT before washing six times with 0.1% PBS-Tween. 4-nitrophenyl phosphate in diethanolamine buffer (Pierce, Loughborough, UK) was added as a substrate and the plates were incubated for 15 min. The absorbance (405 nm) was measured using an ELx800 microplate reader (Cole Parmer, London, UK).

### Proliferation assay

T-cell responses were assayed using the CellTrace Violet Proliferation assay, as described elsewhere (31). Not all individuals were included in this assay because of cell availability: pre-Vx1, n = 16; post-Vx1, n = 15; pre-Vx2, n = 12; post-Vx2, n = 11. Cryopreserved PBMCs were thawed in 30 mL RPMI containing 10% human AB serum (Sigma), 2 mM L-Glut and 1 mM Pen-Strep. Cells were washed twice with PBS and stained with CellTrace Violet (Life Technologies) at 2.5 uM for 10 min at RT. Cold FCS was added to quench the reaction. Cells were plated at 250,000 cells per well in a 96-well round-bottom plate. Peptide pools covering SARS-CoV-2 S1, S2, M, and N, as well as HCoV-OC43 and HCoV-HKU1 S, were added to stimulate cells at a final concentration of 1 ug/ml (Mimotopes, USA) (**Supplementary Table 1**). Media containing 0.1% DMSO (Sigma) was used as a negative control. Phytohaemagglutinin L (Sigma) was used as the positive control at a final concentration of 2 ug/ml. Plates were incubated at 37°C, 5% CO2, and 95% humidity for 7 days, with a hemimedia change on day 4. On day 7, the cells were washed in PBS and stained with fluorochrome-conjugated antibodies against CD4, CD8, and CD3 in PBS. LIVE/DEAD Fixable Aqua was used as a viability marker (Thermo Fisher Scientific). Cells were fixed in 4% paraformaldehyde (Sigma) for 10 min at 4°C and washed in PBS before being stored at 4°C in the dark before being run on MACSquant X (Miltenyi). The gating strategy is illustrated in **Supplementary Figure 6**.

### Statistical analysis

All analyses were performed using the GraphPad Prism 9.0. For pairwise comparisons, two-tailed Mann–Whitney tests were used for unpaired data and Wilcoxon signed-rank tests for paired data. Spearman rank tests were used for correlations.

## Data availability statement

The raw data supporting the conclusions of this article will be made available by the authors, without undue reservation.

## Ethics statement

The studies involving humans were approved by University of Oxford Central University Research Ethics Committee. The studies were conducted in accordance with the local legislation and institutional requirements. Written informed consent for participation in this study was provided by the participants’ legal guardians/next of kin.

## Author contributions

CJ: Conceptualisation, Formal analysis, Investigation, Writing—Original Draft Preparation, Review & Editing, Visualisation. EA: Investigation, Data Curation, Writing—Review & Editing, Project administration. AC: Investigation, Data Curation, Writing—Review & Editing, Project administration. NL: Investigation, Writing—Review & Editing. SL: Formal analysis, Investigation, Writing—Review & Editing. AO: Investigation, Writing—Review & Editing. JR: Writing—Review & Editing. OS: Investigation, Writing—Review & Editing. PITCH Consortium: Investigation, Formal analysis, Data Curation. CT: Resources, Writing—Review & Editing, Methodology. EB: Conceptualisation, Writing—Review & Editing. SD: Supervision, Writing—Review & Editing. LT: Supervision. PK: Supervision, Writing—Review & Editing, Conceptualisation, Methodology. MC: Supervision, Writing—Review & Editing, Methodology. PG: Conceptualisation, Methodology, Resources, Writing—Review & Editing, Supervision, Project administration, Funding acquisition. All authors contributed to the article and approved the submitted version.

## Supplementary material

Supplementary Figure 1 Fold change in nAb and IgG titre in adolescents post-Vx1 and post-Vx2. Fold change in nAbs targeting S **(A)** and RBD **(B)** in infection-naive adolescents (grey circles), and previously-infected adolescents (red circles) post-Vx1 and post-Vx2 as measured by an MSD ACE2-S binding immunoassay. Fold change in IgG targeting S **(D)** and RBD **(D)** in infection-naive and previously-infected adolescents as measured by an MSD v-plex immunoassay. P-values represent Mann-Whitney test values.

Supplementary Figure 2 nAb responses to SARS-CoV-2 variants. Percent inhibition of ACE2-S binding for common variants in infection-naïve (grey) and previously-infected (red) adolescents. P-values from Wilcoxon tests.

Supplementary Figure 3 Cellular responses to endemic HCoVs. Proliferating CD4+ and CD8+ T-cells targeting HCoV-OC43 S2 **(A)** and HCoV-HKU1 S2 **(B)** in infection-naïve (grey) individuals. % proliferating CD4+ and CD8+ T-cells targeting HCoV-OC43 S2 **(C)** and HCoV-HKU1 S2 **(D)** in previously-infected individuals (red). Values below 1% were given nominal values of 0.9%.

Supplementary Figure 4 Cellular responses to M and N antigens. Proliferating CD4+ and CD8+ T-cells targeting M **(A)** and N **(B)** in infection-naïve (grey) and previously-infected (red) adolescents. Values below 1% were given nominal values of 0.9%.

Supplementary Figure 5 IgG responses to endemic HCoVs. IgG targeting SARS-CoV-1, MERS-CoV, HCoV-HKU1, HCoV-OC43, HCoV-NL63 and HCoV-229E in infection-naïve (grey) and previously-infected (red) individuals **(A)**. Correlation between HKU1 **(C)** and OC43 **(D)** to SARS-CoV-2 IgG ratio and nAb response in infection-naïve (grey) and previously-infected (red) individuals.

Supplementary Figure 6 Gating strategy for T-cell proliferation assay. Gates were drawn on lymphocytes (SSC-A × FSC-A), single cells (FSC-H × FSC-A), live cells (CD3 × Live/Dead stain), and CD4+ and CD8+ cells (CD4 × CD8). Proliferating CD4+ cells were gated as compared to a negative control (CD4 × CTV). Proliferating CD8+ cells were gated as compared to a negative control (CD8 × CTV).

Supplementary Table 1 List of peptides used for T-cell assays.

Supplementary Table 2 Full author list for PITCH Consortium.
