# Supplementary figures and images for: Age- and sex-specific differences in immune responses to BNT162b2 COVID-19 and live-attenuated influenza vaccines in UK adolescents
Source: Front Immunol. 2023 Oct 6;14:1248630. doi: 10.3389/fimmu.2023.1248630 (PMC10627794; doi:10.3389/fimmu.2023.1248630)

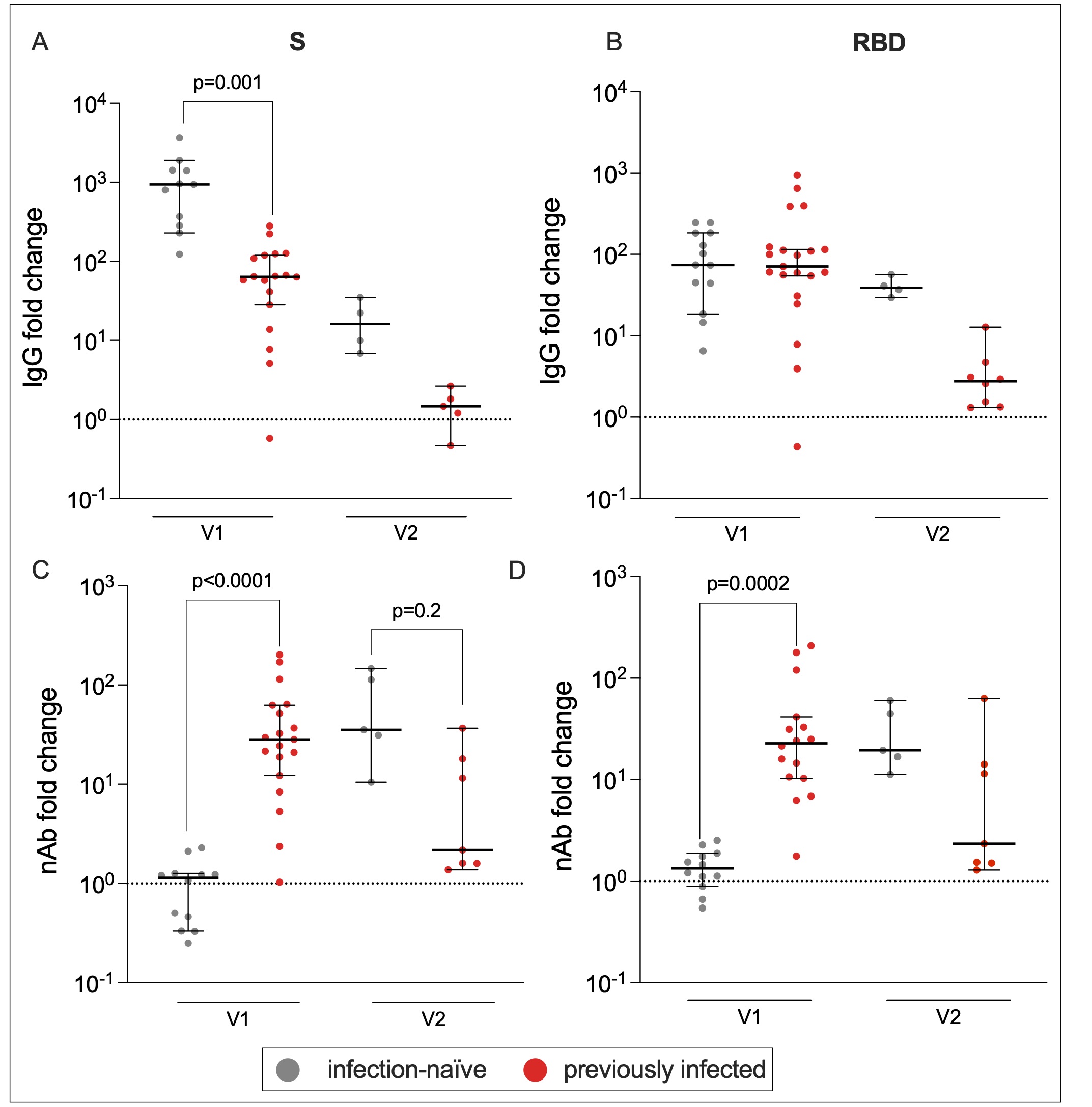

Supplement: Supplementary Figure 1 — Fold change in nAb and IgG titre in adolescents post-Vx1 and post-Vx2. Fold change in nAbs targeting S (A) and RBD (B) in infection-naive adolescents (grey circles), and previously-infected adolescents (red circles) post-Vx1 and post-Vx2 as measured by an MSD ACE2-S binding immunoassay. Fold change in IgG targeting S (D) and RBD (D) in infection-naive and previously-infected adolescents as measured by an MSD v-plex immunoassay. P-values represent Mann-Whitney test values. [file Image_1.jpeg]

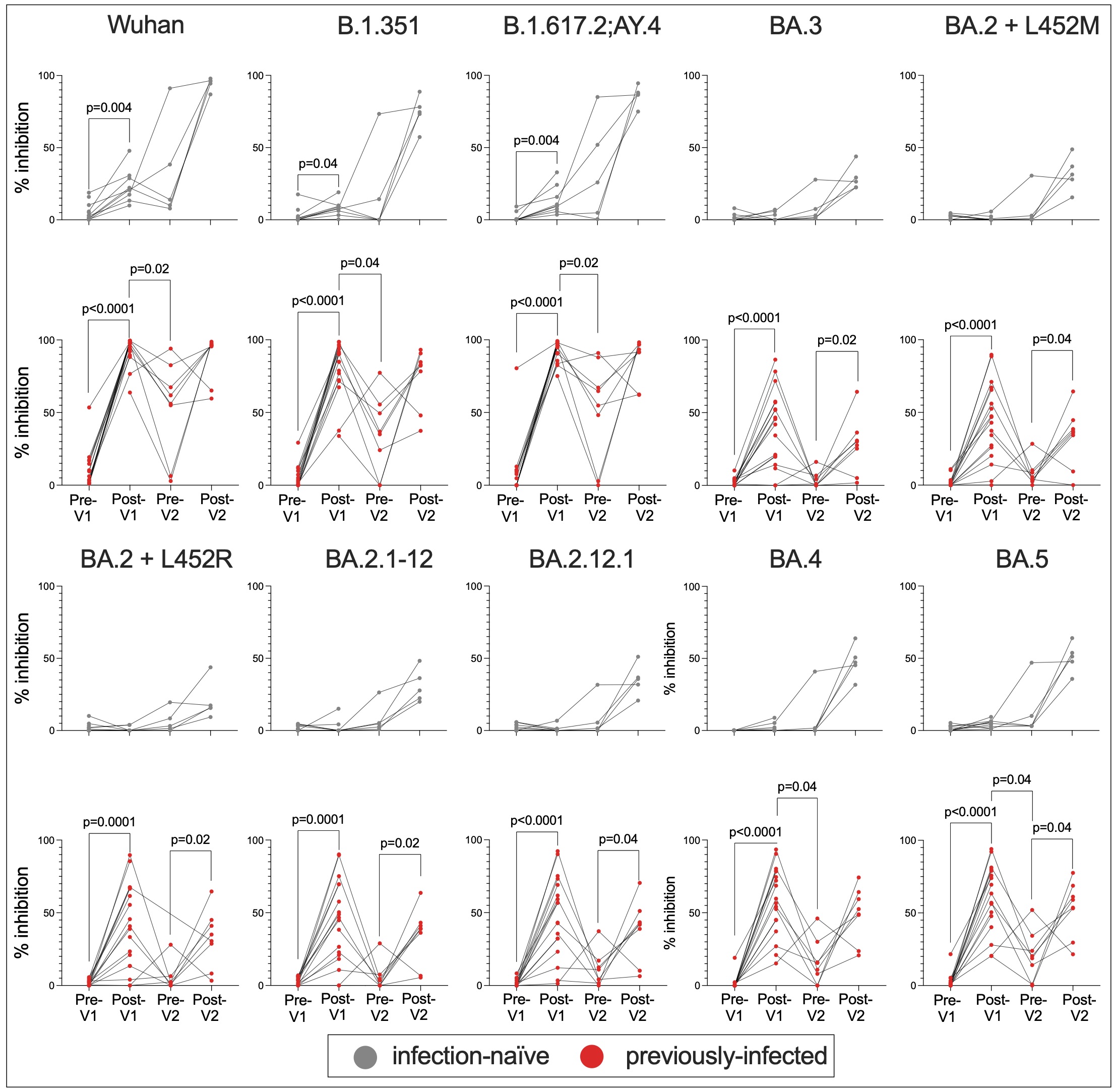

Supplement: Supplementary Figure 2 — nAb responses to SARS-CoV-2 variants. Percent inhibition of ACE2-S binding for common variants in infection-naïve (grey) and previously-infected (red) adolescents. P-values from Wilcoxon tests. [file Image_2.jpeg]

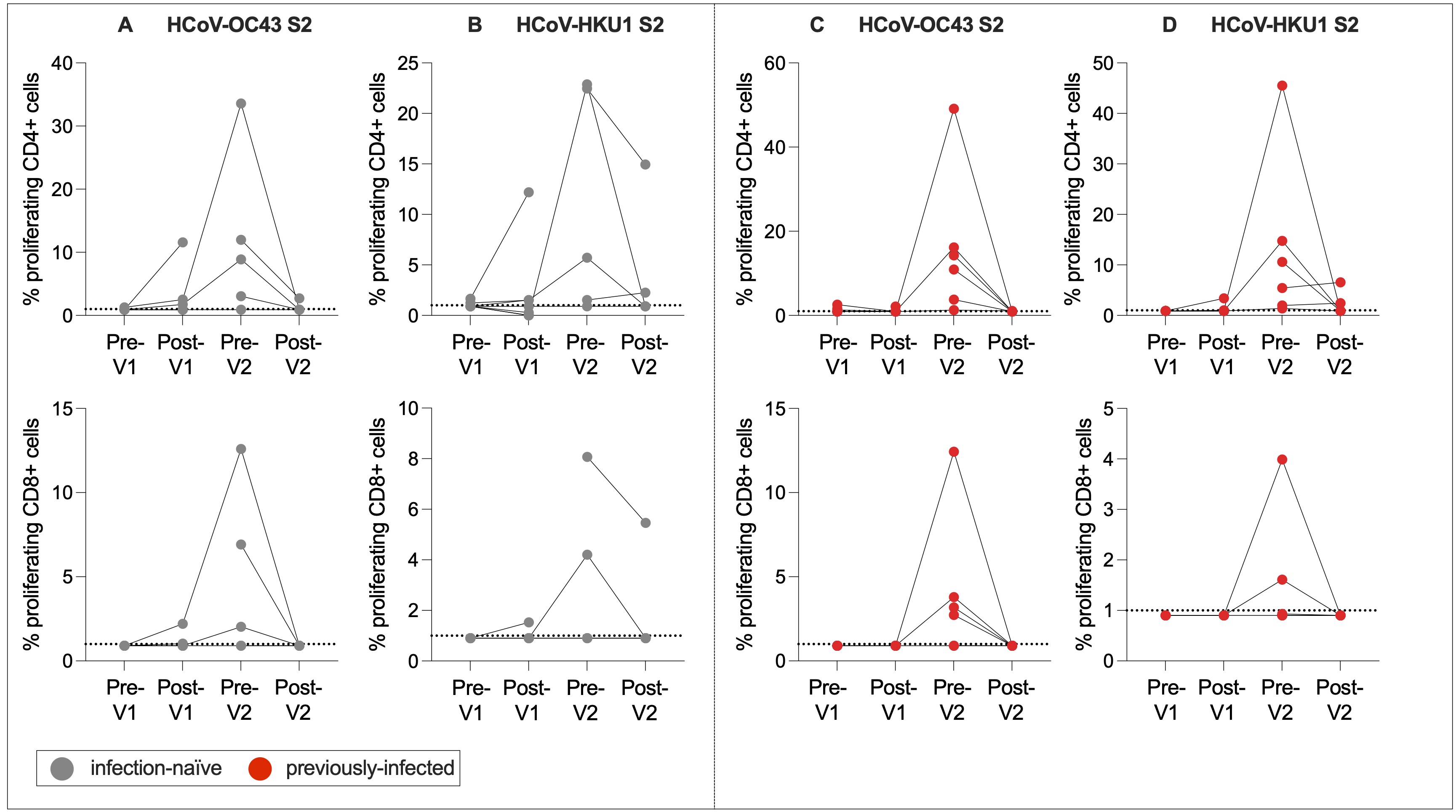

Supplement: Supplementary Figure 3 — Cellular responses to endemic HCoVs. Proliferating CD4+ and CD8+ T-cells targeting HCoV-OC43 S2 (A) and HCoV-HKU1 S2 (B) in infection-naïve (grey) individuals. % proliferating CD4+ and CD8+ T-cells targeting HCoV-OC43 S2 (C) and HCoV-HKU1 S2 (D) in previously-infected individuals (red). Values below 1% were given nominal values of 0.9%. [file Image_3.jpeg]

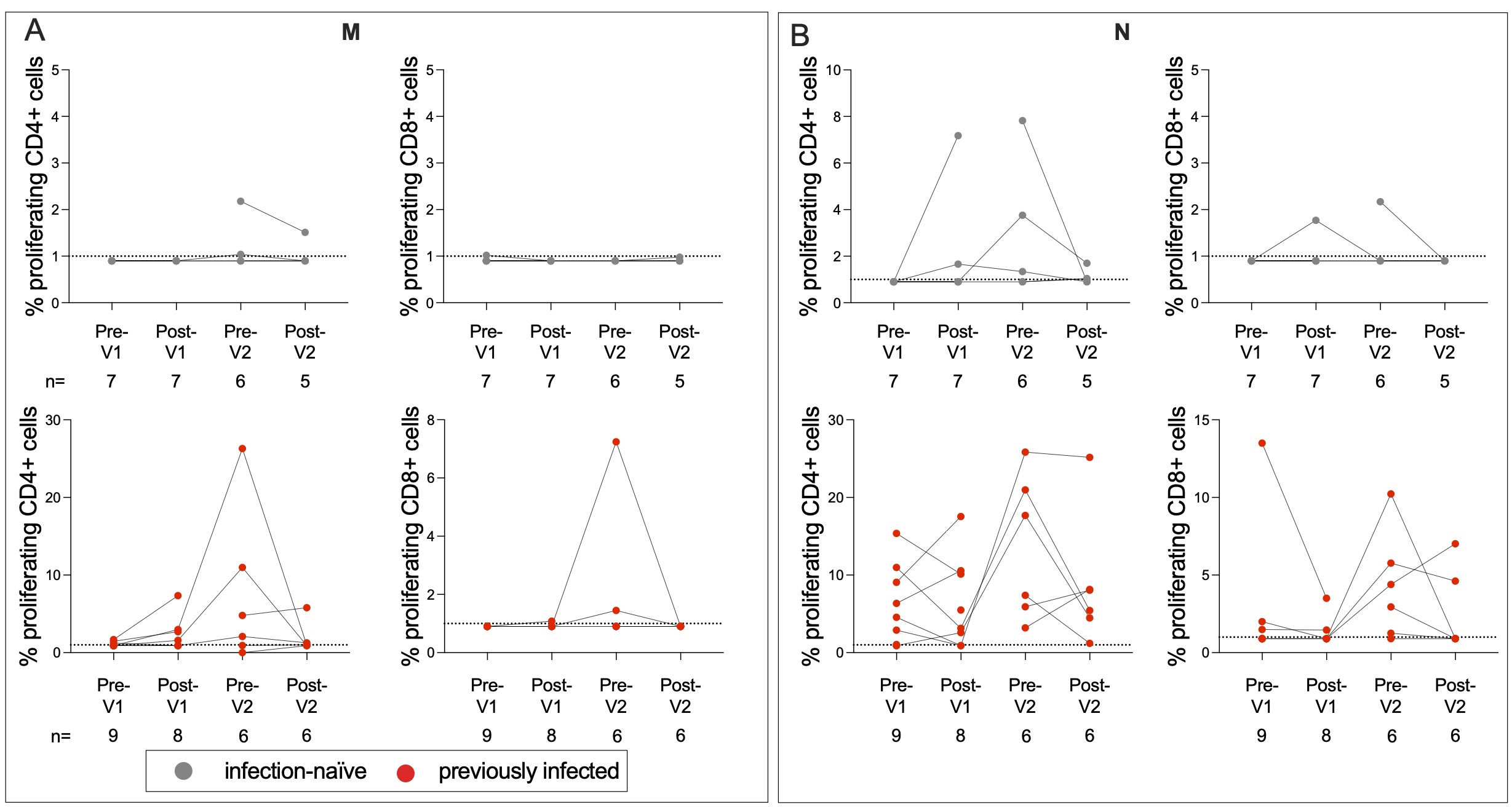

Supplement: Supplementary Figure 4 — Cellular responses to M and N antigens. Proliferating CD4+ and CD8+ T-cells targeting M (A) and N (B) in infection-naïve (grey) and previously-infected (red) adolescents. Values below 1% were given nominal values of 0.9%. [file Image_4.jpeg]

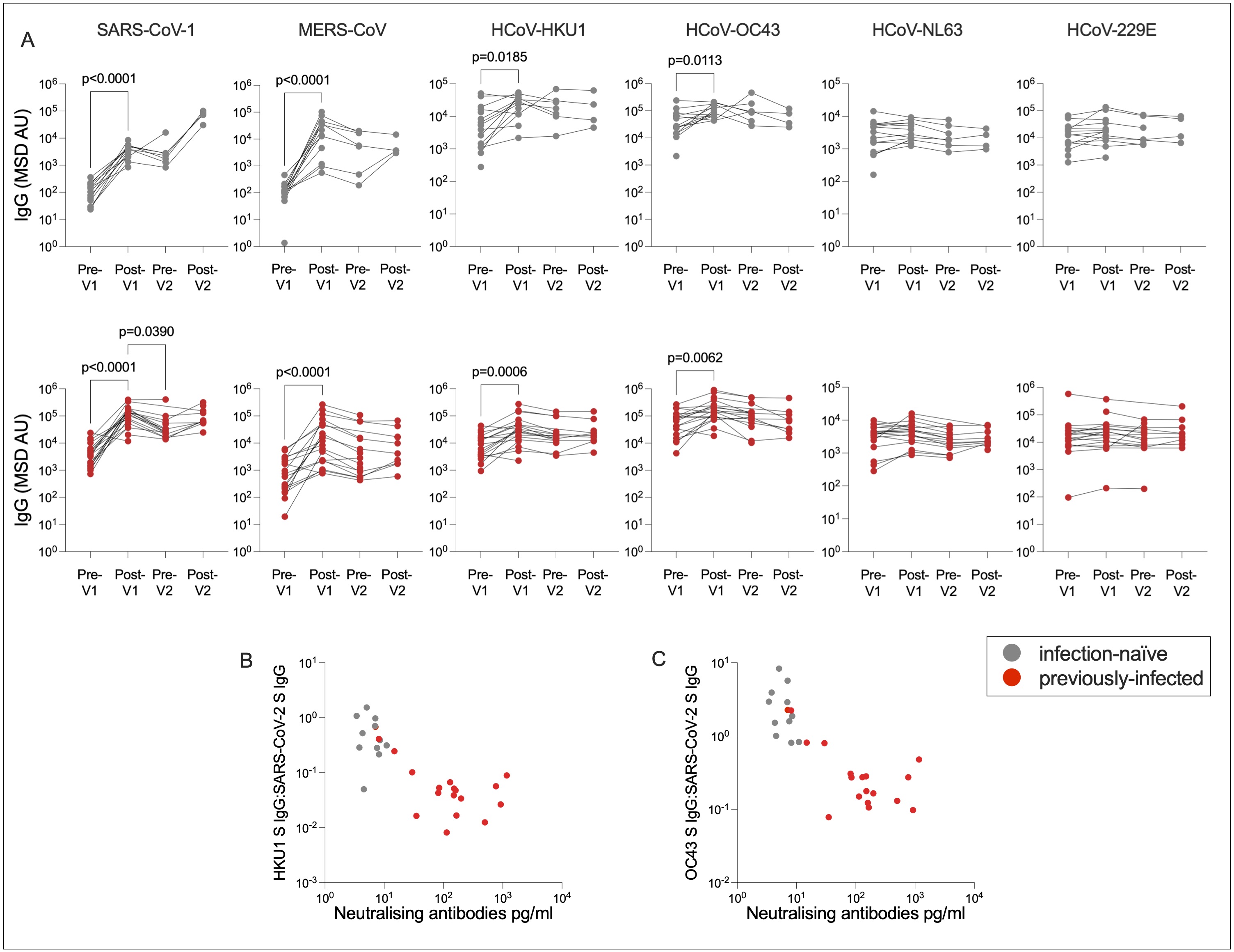

Supplement: Supplementary Figure 5 — IgG responses to endemic HCoVs. IgG targeting SARS-CoV-1, MERS-CoV, HCoV-HKU1, HCoV-OC43, HCoV-NL63 and HCoV-229E in infection-naïve (grey) and previously-infected (red) individuals (A). Correlation between HKU1 (C) and OC43 (D) to SARS-CoV-2 IgG ratio and nAb response in infection-naïve (grey) and previously-infected (red) individuals. [file Image_5.jpeg]

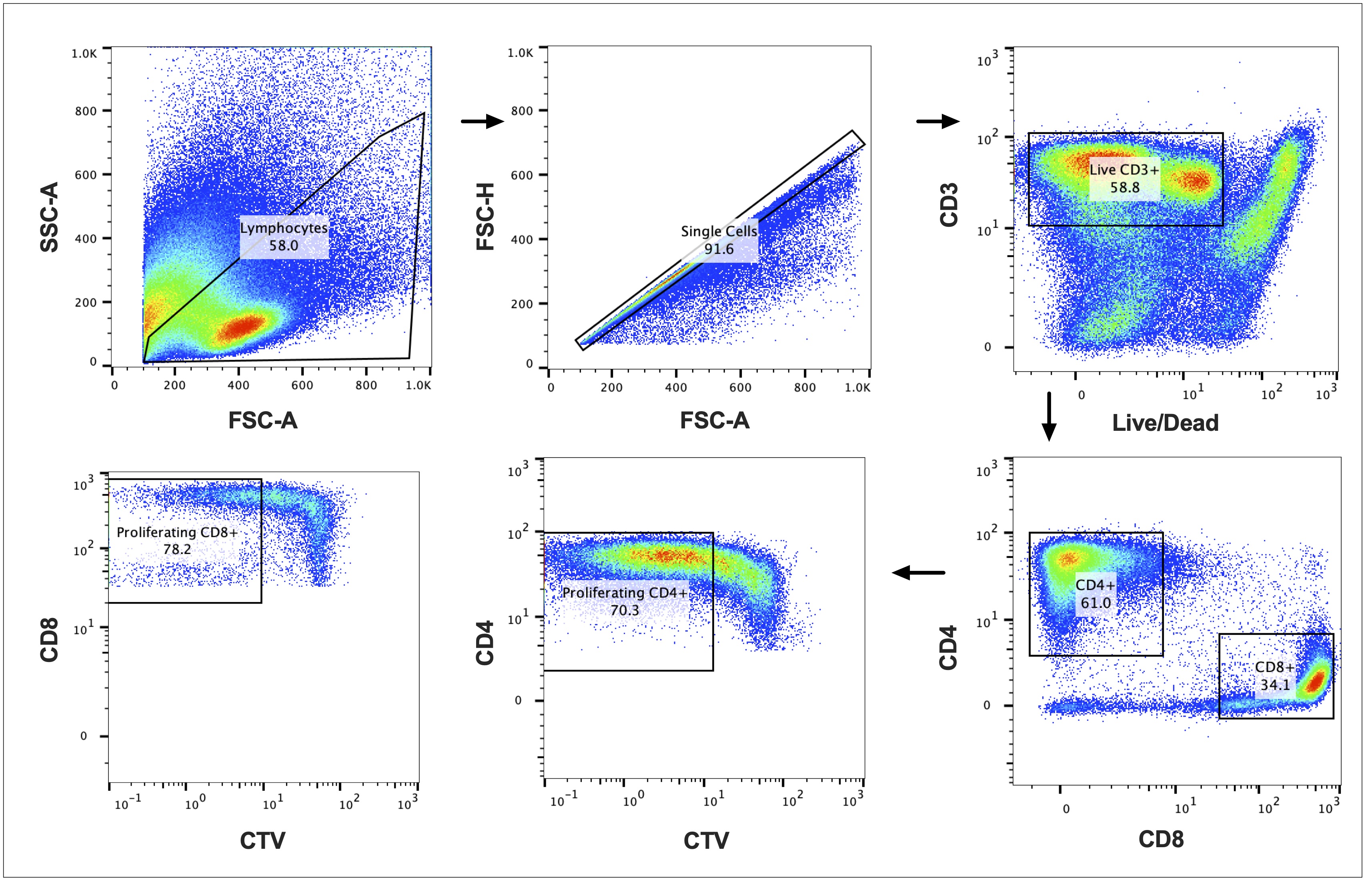

Supplement: Supplementary Figure 6 — Gating strategy for T-cell proliferation assay. Gates were drawn on lymphocytes (SSC-A × FSC-A), single cells (FSC-H × FSC-A), live cells (CD3 × Live/Dead stain), and CD4+ and CD8+ cells (CD4 × CD8). Proliferating CD4+ cells were gated as compared to a negative control (CD4 × CTV). Proliferating CD8+ cells were gated as compared to a negative control (CD8 × CTV). [file Image_6.jpeg]
